# Adjuvant therapy in chromophobe renal cell carcinoma: current evidence and unmet needs

**DOI:** 10.3389/fonc.2026.1910114

**Published:** 2026-09-03

**Authors:** Niovi Bejjani, Joseph Kattan

**Affiliations:** Department of Hematology-Oncology, University Medical Center Hotel Dieu de France, Faculty of Medicine, Saint Joseph University of Beirut, Beirut, Lebanon

**Keywords:** adjuvant therapy, chromophobe renal cell carcinoma, immunotherapy, mTOR inhibitors, non-clear cell RCC, VEGFR tyrosine kinase inhibitors

## Abstract

**Objectives:**

Chromophobe renal cell carcinoma (chRCC) is a rare subtype of kidney cancer with biological and clinical features that differ significantly from clear cell renal cell carcinoma (ccRCC). While adjuvant treatments have evolved for ccRCC, chRCC has been excluded or understudied in clinical trials, which has led to insufficient histology-specific data to guide postoperative treatment. This research aimed to summarize and critically appraise the current evidence regarding adjuvant therapies for chRCC, highlight existing limitations in the literature, and identify priorities for future research.

**Methods:**

A literature review was performed using PubMed and Google Scholar with search terms including “Carcinoma, Renal cell, Chromophobe, RCC, adjuvant, treatment, postoperative therapy, kidney cancer”. The search was limited to the last 10 years and included various study designs, ranging from phase III trials to case reports, that specifically addressed chRCC rather than grouping it broadly under non-clear cell RCC.

**Results:**

Current clinical evidence does not support the routine use of adjuvant systemic therapies in chRCC. The phase III EVEREST trial failed to demonstrate a statistically significant improvement in recurrence-free survival for the mTOR inhibitor everolimus in the chRCC subgroup. Similarly, the ECOG ACRIN E2805 trial found that adjuvant VEGFR-targeted therapies, such as sunitinib and sorafenib, resulted in excessive toxicity without providing a disease-free survival benefit over placebo. Data on immune checkpoint inhibitors like pembrolizumab and nivolumab remain limited to metastatic settings with low objective response rates, resulting in insufficient evidence and a lack of expert consensus for their adjuvant use in chRCC.

**Conclusion:**

There is a substantial lack of evidence supporting any reliable adjuvant therapy for chRCC, leaving high-risk patients without proven options after nephrectomy, and questioning the consistency of the extrapolation from metastatic clear-cell populations. However, given the rarity of chRCC and its comparatively low recurrence risk, a dedicated adjuvant randomized trial may be neither statistically nor logistically feasible; priorities should instead include first establishing an effective systemic therapy in the metastatic setting, pursuing pooled non–clear cell or registry-based collaborations, and developing biomarker-driven patient selection.

## Introduction

Chromophobe renal cell carcinoma (chRCC) is a rare subtype of non-clear cell renal cell carcinoma (nccRCC), accounting for approximately 5-7% of all renal cell carcinomas (RCC) (1), while clear cell type represents 75–85% of primary kidney malignancies. The nccRCC comprises a heterogeneous group of tumors, including next to chromophobe, papillary, collecting duct, translocation-associated, medullary, and unclassified subtypes (2, 3). These entities differ significantly in their molecular drivers, clinical behavior, and therapeutic responsiveness from those of clear cell renal cell carcinoma (ccRCC) phenotypes (2).

Localized RCC is primarily managed with partial or radical nephrectomy with curative intent. However, disease recurrence occurs in approximately 25-50% of all localized RCC patients, depending on risk stratification (4). Although chRCC has been reported to have a more favorable prognosis than ccRCC, some studies have described a more aggressive clinical course, with recurrence rates ranging from 16% to 30% (5). In contrast, surgical series reported disease-specific events or recurrences in only approximately 4-6% of patients at 5 years and 6-11% at 10 years with chRCC (6, 7). This risk is highly concentrated in a small subset of patient with chRCC with adverse pathological features: sarcomatoid differentiation and lymph node involvement confer a markedly higher risk (reported 10-year recurrence-free survival of roughly 34% versus 91% in their absence), as do higher tumor stage (≥ pT2b), coagulative tumor necrosis, larger tumor size, small-vessel invasion, and positive surgical margins (6–8). Conversely, the majority of patients; those with small, organ-confined, low-grade tumors lacking these features, are at very low risk of relapse (6–8).

Adjuvant therapies aim to eradicate residual microscopic disease and prevent metastatic progression. Data from the metastatic setting with mTOR inhibitors, VEGFR-targeted tyrosine kinase inhibitors, and immune checkpoint inhibitors have largely guided the design and direction of adjuvant therapy trials, raising interest in the utility of these agents in the adjuvant setting (9).

Adjuvant treatment strategies for ccRCC have evolved considerably in recent years, although randomized trials have yielded mixed results. Among VEGFR-targeted agents, sunitinib improved disease-free survival in the S-TRAC trial (10), whereas sorafenib, pazopanib, and axitinib (ASSURE, PROTECT, ATLAS, and SORCE) showed no adjuvant benefit (11–14). Adjuvant immune checkpoint inhibition has likewise been largely negative (IMmotion010, CheckMate 914, and the perioperative PROSPER trial) (15–17), with the notable exception of pembrolizumab in KEYNOTE-564, which improved disease-free survival and remains the only adjuvant RCC trial to demonstrate a significant overall survival benefit (hazard ratio 0.62) (18, 19). More recently, the randomized phase 3 LITESPARK-022 study, demonstrated that the addition of belzutifan, a hypoxia-inducible factor 2a (HIF-2a inhibitor), to Pembrolizumab shows higher benefit with improved disease-free survival compared to Pembrolizumab alone in adjuvant setting in patients with ccRCC (20). Of the adjuvant trials reported to date, the RAMPART study (evaluating durvalumab with or without tremelimumab) is one of the few to enroll both clear cell and non–clear cell histologies, including chRCC. While the combination improved disease-free survival in the overall population, efficacy data for non–clear cell subtypes, including chromophobe RCC, remain immature, precluding definitive conclusions regarding its benefit in these subgroups (21).

Most clinical trials in RCC have predominantly focused on ccRCC, often grouping nccRCC subtypes together or excluding them entirely. Therefore, chRCC remains underrepresented in clinical research, particularly in the adjuvant setting. As a result, there is an unmet need data to guide adjuvant treatment decisions in patients with chRCC. Furthermore, much of the available evidence is extrapolated from metastatic settings, limiting its applicability to the postoperative context.

This review aims to summarize and critically appraise the current evidence on adjuvant therapies in chRCC, highlight key limitations in literature, and identify areas for future research.

## Biological and clinical specificities of chromophobe RCC

ChRCC exhibits distinct biological features compared to ccRCC, including frequent alterations in the mTOR pathway that correlate with poor prognosis and potential sensitivity to mTOR inhibitors (22). Germline Folliculin (FLCN) mutations, a tumor suppressor gene, associated with Birt-Hogg-Dubé syndrome, predispose to chRCC often presenting as hybrid oncocytic/chromophobe tumors, although they account for only a small proportion of cases and should not be regarded as a universal or defining feature of the subtype (22, 23). These FLCN-tumors have a mTOR pathway upregulation reinforcing the idea that chRCC may be a real target for mTOR inhibitors (24). These tumors also show differing responses to VEGF-targeted therapies and immunotherapy compared to ccRCC, with more limited efficacy observed in systemic treatments (25). Sporadic chRCC, which represents the vast majority of cases, was characterized by recurrent alterations involving TP53 (~33%) and PTEN (~9%) in the largest genomic characterization to date (n = 66) (26). These differences may explain the limited efficacy of therapies developed for ccRCC when applied to chRCC.

## Literature search strategy

This narrative review was informed by a structured search of PubMed and Google Scholar using combinations of the terms “chromophobe renal cell carcinoma”, “chromophobe RCC”, “non-clear cell RCC”, “adjuvant”, “postoperative therapy”, “nephrectomy”, “mTOR inhibitor”, “VEGFR tyrosine kinase inhibitor”, and “immune checkpoint inhibitor”. The search was restricted to articles published within the last ten years; this window was chosen deliberately, as the first adjuvant VEGFR-tyrosine kinase inhibitor trials began reporting from 2016 onward, and it therefore captures the modern era of adjuvant RCC therapy.

Eligible study designs ranged from phase III randomized trials to phase II studies, retrospective cohorts, case series, and case reports. Studies reporting chromophobe RCC as a distinct histology, or as an identifiable subgroup, were prioritized over those pooling it indiscriminately under non-clear cell RCC. Studies addressing only the surgical management of chRCC without systemic adjuvant therapy were excluded, as were studies from which chromophobe-specific data could not be extracted. Because evidence specific to the adjuvant setting is scarce, selected metastatic-setting studies were included solely to provide biological and clinical context and are identified as such throughout. As a narrative rather than a systematic review, this synthesis did not follow a formal PRISMA protocol, and no formal risk-of-bias assessment was undertaken, a limitation shared by the sparse underlying evidence base.

## Current evidence for adjuvant systemic therapy in chRCC

The adjuvant systemic therapies evaluated in RCC are summarized in **Table 1**, which shows that chromophobe RCC has been either excluded from or represented by only small numbers within these trials, and that almost all adjuvant trials have been negative. Studies providing chromophobe-specific efficacy data (spanning both the adjuvant and metastatic settings) are summarized in **Table 2**. The sections that follow focus on the adjuvant evidence; data from the metastatic setting are addressed separately and only for context.

**Table 1:** Adjuvant systemic therapy trials in renal cell carcinoma and representation of chromophobe histology.

Trial	Agent (class)	Total N	Histology eligible	chRCC incl. (n)	Primary endpoint: result	Ref
ASSURE (E2805)	Sunitinib/sorafenib (VEGFR-TKI)	1,943	Clear + non-clear	Yes (111)	DFS: negative	(12)
S-TRAC	Sunitinib (VEGFR-TKI)	615	Clear cell only	No	DFS: positive; no OS benefit	(10)
PROTECT	Pazopanib (VEGFR-TKI)	1,538	Clear cell only	No	DFS: negative	(11)
ATLAS	Axitinib (VEGFR-TKI)	724	Clear cell only	No	DFS: negative	(14)
SORCE	Sorafenib (VEGFR-TKI)	1,711	Clear + non-clear	Yes (96)	DFS: negative	(13)
EVEREST (S0931)	Everolimus (mTOR)	1,545	Clear + non-clear	Yes (99)	RFS: negative overall	(27, 28)
KEYNOTE-564	Pembrolizumab (PD-1)	994	Clear cell only	No	DFS positive; OS positive (HR 0.62)	(18, 19)
IMmotion010	Atezolizumab (PD-L1)	778	Clear cell/sarcomatoid	No	DFS: negative	(17)
CheckMate 914	Nivolumab + ipilimumab	816	Clear cell only	No	DFS: negative	(16)
PROSPER	Perioperative nivolumab	819	Clear + non-clear	Yes (44)	RFS: negative	(15)
RAMPART	Durvalumab ±	~790	Clear + non-clear	Yes (~6%)	DFS/OS: ongoing	(21)
LITESPARK-022	Pembrolizumab + belzutifan	1,841	Clear cell only	No	DFS: positive	(20)

DFS, disease-free survival; RFS, recurrence-free survival; OS, overall survival; chRCC, chromophobe RCC.

**Table 2:** Summary of key studies providing chromophobe-specific data.

Study (year)	Design	Setting	chRCC (n)	Intervention	Outcome in chRCC	Major limitations	Ref
EVEREST/Gulati (2024)	Phase III secondary analysis	Adjuvant	99	Everolimus vs placebo	RFS HR 0.89, non-significant	Small subgroup; not powered for histology	(28)
E2805/Haas (2016)	Phase III RCT	Adjuvant	111	Sunitinib/sorafenib vs placebo	No DFS benefit; excess toxicity	Histology-mixed; not subtype-powered	(12)
Koh (2013)	Phase II	Metastatic	8	Everolimus	ORR 25%; PFS 13.1 mo; OS 21.6 mo	Very few chRCC cases	(29)
Koshkin (2018)	Retrospective	Metastatic	5	Nivolumab	No objective responses; some stable disease	Retrospective; few chRCC	(30)
KEYNOTE-427/McDermott (2021)	Phase II	Metastatic	21	Pembrolizumab	ORR 9.5%	Small subgroup	(31)
Hutson (2021)	Phase II	Metastatic	9	Lenvatinib + everolimus	ORR 44% (chromophobe subset)	Very small n	(32)
Voss (2016)	Phase II	Metastatic	5	Everolimus + bevacizumab	Limited disease control	Small n	(33)
Lee (2022)	Phase II	Metastatic	7	Cabozantinib + nivolumab	No objective responses; DCR 71% (stable disease)	Early closure; very small n	(34)
Grünwald (2025)	Delphi consensus	Adjuvant	–	Pembrolizumab (expert opinion)	Low consensus for adjuvant use in chRCC	Expert opinion, not trial data	(35)

ORR, objective response rate; PFS, progression-free survival; OS, overall survival; DCR, disease control rate; RFS, recurrence-free survival; HR, hazard ratio.

### Role of mTOR inhibition (everolimus) in the adjuvant setting in chRCC

The use of everolimus, an inhibitor of the mammalian target of rapamycin (mTOR) pathway, is supported by a biological rationale in chRCC, which frequently exhibits alterations leading to mTOR pathway activation, including mutations associated with FLCN dysfunction. This has led to interest in evaluating mTOR inhibition as a therapeutic strategy in this subtype (26, 36).

The phase III EVEREST trial (Gulati et al., 2024) represents the largest study to date assessing adjuvant everolimus in patients with RCC at intermediate or high risk of recurrence following radical or partial nephrectomy. Among the study population, 99 patients had chromophobe histology (53 received everolimus vs 46 took placebo). The trial failed to demonstrate a statistically significant improvement in recurrence-free survival or overall survival with adjuvant everolimus compared with placebo. Moreover, treatment was associated with a significantly higher incidence of grade 3 or higher adverse events. Although the lower bound of the confidence interval for recurrence-free survival approached unity (0.89), suggesting a possible modest benefit in select subgroups, these findings were not sufficient to support routine use (28).

Overall, while there is a sound biological rationale for targeting the mTOR pathway in chromophobe RCC, current clinical evidence does not support the use of everolimus in the adjuvant setting. The available data are limited by small sample sizes, heterogeneity of study populations, and reliance on metastatic cohorts, underscoring the need for dedicated, histology-specific trials. The limited metastatic everolimus data are included in **Table 2** and discussed further below.

### Role of anti-VEGF in adjuvant setting in chRCC

The ECOG ACRIN E2805 study evaluated adjuvant sunitinib and sorafenib in patients with high-risk, non-metastatic RCC. Among the 111 patients with chRCC, 40 received sunitinib, 43 sorafenib, and 28 placebo. The trial showed excessive toxicity despite dose reduction and did not provide results specific to any subtype. Interestingly, disease-free survival favored placebo in group A (sunitinib vs. placebo) and sorafenib in group B (sorafenib vs. placebo). This was the first trial to investigate VEGFR inhibitors as adjuvant therapy in locally advanced, high-risk kidney cancer. The study population was histologically diverse and representative, with overall outcomes better than expected, providing reassurance that the results are relevant to the population at large. However, the median time to disease recurrence did not differ between those who received sorafenib or sunitinib after surgery and those who received the placebo (12).

A significant concern is that many patients in this trial likely remained disease-free but were exposed to toxic effects without benefit. Moreover, patients who experienced recurrence did not benefit from adjuvant therapy and may have compromised their future benefit to VEGFR inhibitors post-relapse. The willingness of these patients to participate deserves recognition, as their contribution informs future generations. Ultimately, the results strongly argue against the use of anti-angiogenic therapy in the adjuvant setting for primary resected renal-cell carcinoma (12). Future research should instead explore other approaches, such as checkpoint inhibitor immunotherapy, ideally within observational or placebo-controlled studies. These findings are consistent with the broader adjuvant VEGFR-TKI experience: S-TRAC, PROTECT, ATLAS, and SORCE collectively failed to establish a role for anti-angiogenic therapy in this setting, and none was powered for chromophobe histology (**Table 1**) (10–14).

### Role of immune checkpoint inhibitors in the adjuvant setting

In contrast to the metastatic setting, dedicated adjuvant data for immune checkpoint inhibitors in chRCC are essentially absent, because the pivotal adjuvant immunotherapy trials were largely restricted to clear cell histology. Pembrolizumab in KEYNOTE-564 improved disease-free survival and is the only adjuvant RCC trial to date to demonstrate a statistically significant overall survival benefit (hazard ratio 0.62); however, the trial enrolled clear cell RCC exclusively and did not include chromophobe patients (18, 19). The other major adjuvant immunotherapy trials were negative: atezolizumab (IMmotion010) and nivolumab plus ipilimumab (CheckMate 914) enrolled clear cell disease only, while the perioperative nivolumab PROSPER trial, which did not permit non-clear cell histologies, was stopped for futility (15–17). The RAMPART trial (adjuvant durvalumab with or without tremelimumab) is an important exception in that it enrolls both clear cell and non-clear cell RCC, including a small series of chromophobe and other nccRCC patients; should a histology-specific secondary analysis become available, it could help clarify the role of checkpoint inhibition in the non-clear cell adjuvant setting (21). Finally, the recently positive LITESPARK-022 trial (pembrolizumab plus belzutifan) was likewise confined to clear cell RCC, so its benefit cannot be assumed to extend to chromophobe disease (20).

In July 2025, a modified Delphi study gathered expert consensus from 15 European diverse RCC specialists; the panel expressed caution regarding pembrolizumab use in nccRCC subtypes, including chRCC, and recommendations to extend adjuvant pembrolizumab to these populations received limited support, reflecting the scarcity of clinical data (35).

## Metastatic systemic therapy: context for the adjuvant setting

Because no systemic agent has established a standard of care in metastatic chRCC, there is by extension no proven therapy to translate into the adjuvant setting; a central reason the adjuvant evidence base is so thin. The metastatic data below, summarized in **Table 2**, are presented only to substantiate this point rather than as guidance for postoperative management.

In a phase II trial by Koh et al., everolimus demonstrated modest activity with an overall response rate (ORR) of 25%, median PFS of 13.1 months, and median OS of 21.6 months in a small subgroup of 8 patients with chRCC (29). In contrast, immune checkpoint inhibitors have shown limited activity as monotherapy, while some patients achieved stable disease, no objective responses were reported with nivolumab in one retrospective cohort with small number of patients with metastatic chRCC (30) and an ORR of 9.5% with pembrolizumab in the chromophobe subgroup (21 patients) of KEYNOTE-427 (31).

Combination strategies have also been explored in small prospective studies. A phase II trial of lenvatinib plus everolimus in advanced nccRCC reported a relatively high ORR (44%) in the chromophobe subgroup, though based on very few patients (32), and everolimus plus bevacizumab produced limited disease control in a minority of advanced chRCC (33). In a phase II study by Lee et al., cabozantinib plus nivolumab was evaluated advanced nccRCC; among seven patients with chromophobe histology and prior nephrectomy, no objective responses were observed. Overall, the study was discontinued early because of limited efficacy, slow accrual and treatment-related toxicity, highlighting the potential risks associated with this therapy (34).

All in all, current evidence is insufficient to establish the efficacy of VEGFR TKI or immune checkpoint inhibitor in advanced or metastatic chRCC. The lack of objective responses, coupled with small sample sizes and reliance on metastatic data, underscores the need for alternative therapeutic strategies tailored to the distinct biology of this subtype. Reinforcing that the absence of an effective metastatic standard leads to no established agent to test in the adjuvant setting.

## Critical analysis of existing data

Overall, the available evidence on adjuvant therapy in chRCC remains limited and difficult to interpret. One of the main issues is that most studies do not analyze chRCC as a distinct entity. Instead, it is usually grouped under nccRCC, often representing only a very small proportion of the study population. This makes it challenging to draw any firm, subtype-specific conclusions.

Another important limitation is that much of the data comes from the metastatic setting. While this can provide useful insights, it does not necessarily translate to the adjuvant context, where the goal is to eliminate microscopic residual disease rather than treat established metastases. As a result, the protective benefit of these therapies after surgery remains uncertain.

From a biological perspective, chromophobe RCC behaves differently from clear cell RCC. It is less angiogenesis-driven and more closely associated with alterations in pathways such as mTOR (22). This may partly explain why treatments that are effective in ccRCC, particularly VEGFR-targeted therapies and immunotherapy, have shown limited activity in this subtype. Even for mTOR inhibitors, where there is a strong theoretical rationale, clinical results have not been convincing in the adjuvant setting.

In addition, many studies are limited by small sample sizes, incomplete reporting (such as prior nephrectomy status), and heterogeneous patient populations. These factors further complicate interpretation and reduce the strength of the conclusions that can be drawn.

Taken together, the current evidence is insufficient to support a reliable adjuvant systemic therapy in chRCC. Thus, our young patients at high risk of relapse after nephrectomy for chRCC remain without a proven adjuvant treatment option.

This unanswered question raises a critical clinical dilemma: do we have the ethical and scientific justification to extrapolate from the adjuvant data for ccRCC and offer Pembrolizumab alone or with Belzutifan to these chRCC? Since the FDA’s 2021 approval (9, 37) announcement for adjuvant pembrolizumab explicitly includes *patients with renal cell carcinoma at intermediate-high or high risk of recurrence following nephrectomy, or following nephrectomy and resection of metastatic lesions*, without precising the exact histology subtype. Yet, the KEYNOTE-564 trial, which secured this approval, was not designed to evaluate non-clear-cell subtypes like chRCC separately, despite demonstrating statistically significant improvement in disease-free survival (19). Beyond the United States, the regulatory picture is similarly permissive in wording but not in evidence: the European Medicines Agency approved adjuvant pembrolizumab for “renal cell carcinoma at increased risk of recurrence” without restricting the indication to clear cell histology (38), and NICE in the United Kingdom recommends pembrolizumab as an option for adjuvant treatment of RCC in those at increased risk of recurrence after nephrectomy (39).

Of note, the recent FDA’s approval announcement (June 12, 2026) of Belzutifan plus Pembrolizumab is clearly limited to clear cell RCC (40). As we stand at this crossroad, the question remains unanswered: should the broad language of the FDA approval guide our practice for chRCC, or should we await histology-specific evidence before exposing vulnerable patients to potential toxicity without guaranteed benefit?

The answer will shape the future of adjuvant management in chRCC, and may determine whether these patients receive hope or remain in therapeutic limbo.

## Future directions

Given the current limitations in the literature, future research should focus on developing studies specifically designed for chRCC rather than continuing to group it with other non–clear cell subtypes. Because of its rarity, this will likely require multicenter and international collaboration to ensure adequate patient numbers.

Any realistic discussion of such trials, however, must begin with the numbers. Chromophobe RCC constitutes only about 5–7% of RCC, and only roughly 5–10% of localized cases recur (1, 6); the genuinely high-risk, resectable population that might be targeted for adjuvant therapy is therefore very small. A conventional randomized adjuvant trial powered to detect even a moderate disease-free survival benefit (for example, under conventional assumptions of a two-sided α of 0.05, 80% power, and a target hazard ratio of 0.70), would require approximately 250 disease-free survival events. The number of patients needed to observe these events would depend on the expected recurrence rate and duration of follow-up but could readily reach several hundred to more than 1,000 patients in a rare, relatively favorable-risk population.

More fundamentally, we do not yet know the optimal systemic therapy for metastatic chRCC, and no agent has convincingly cleared the efficacy bar in that setting. It is therefore difficult to justify launching adjuvant studies of agents whose activity in established chromophobe disease is unproven. Defining effective therapy in the metastatic setting should logically precede and inform any adjuvant effort.

Where trials are pursued, pragmatic designs are more realistic than a stand-alone chromophobe RCT. Adaptive and platform trials, in which several rare non-clear cell histologies share a common control arm and infrastructure, with pre-specified, histology-specific analyses, offer a way to study chRCC without demanding an impractical dedicated sample size.

Overall, a shift toward more tailored and collaborative research efforts will be essential to address the current gaps in evidence.

## Conclusion

In summary, there is a substantial lack of evidence supporting the use of adjuvant therapies in chromophobe RCC. Available data, largely derived from small and heterogeneous cohorts or extrapolated from metastatic settings, do not demonstrate a clear survival benefit and are often associated with significant toxicity. The distinct biological profile of chRCC further underscores the need for subtype-specific therapeutic strategies. Rather than reflexively calling for dedicated randomized trials; which may be infeasible in so rare and comparatively indolent a subtype, future efforts should first aim to establish an effective systemic therapy in metastatic chRCC, leverage pooled non-clear cell platform designs, biomarker-driven selection, and international registries, and exercise caution when extrapolating clear cell adjuvant data to individual chRCC patients.

## References

[1] Battle D Griffith M Msaouel P Voss MH Staehler MD . Real world data on treatment of chromophobe renal cell carcinoma. J Clin Oncol. (2024) 42:409–. doi: 10.1200/JCO.2024.42.4_suppl.409

[2] Giles RH Choueiri TK Heng DY Albiges L Hsieh JJ Linehan WM . Recommendations for the management of rare kidney cancers. Eur Urol. (2017) 72:974–83. doi: 10.1016/j.eururo.2017.06.040 28720391

[3] Patard J-J Leray E Rioux-Leclercq N Cindolo L Ficarra V Zisman A . Prognostic value of histologic subtypes in renal cell carcinoma: a multicenter experience. J Clin Oncol. (2005) 23:2763–71. doi: 10.1200/JCO.2005.07.055 15837991

[4] Bueno AN Stein MN Runcie K . Adjuvant therapy in renal cell carcinoma (RCC): progress, at last. Transl Cancer Res. (2024) 13:6448-62. doi: 10.21037/tcr-23-2247 39697753 PMC11651811

[5] Armas-Alvarez AL Alois Osorio-Manyari A Donate-Moreno MJ Vera-Beron R Salinas-Sánchez AS . Local and distant recurrence of the chromophobe renal cell carcinoma. Arch Esp Urol. (2020) 73:71–5. 31950927

[6] Neves JB Vanaclocha Saiz L Abu-Ghanem Y Marchetti M Tran-Dang M-A El-Sheikh S . Pattern, timing and predictors of recurrence after surgical resection of chromophobe renal cell carcinoma. World J Urol. (2021) 39:3823–31. doi: 10.1007/s00345-021-03683-9 33851271

[7] Przybycin CG Cronin AM Darvishian F Gopalan A Al-Ahmadie HA Fine SW . Chromophobe renal cell carcinoma: a clinicopathologic study of 203 tumors in 200 patients with primary resection at a single institution. Am J Surg Pathol. (2011) 35:962–70. doi: 10.1097/PAS.0b013e31821a455d 21602658

[8] Pieretti AC Westerman ME Childs A Millward N Shapiro DD Sircar K . Sarcomatoid features and lymph node-positive disease in chromophobe renal cell carcinoma. Urol Oncol. (2021) 39:790.e17–790.e23. doi: 10.1016/j.urolonc.2021.06.016 34301458

[9] Fitzgerald KN Motzer RJ Lee C-H . Adjuvant therapy options in renal cell carcinoma— targeting the metastatic cascade. Nat Rev Urol. (2023) 20:179–93. doi: 10.1038/s41585-022-00666-2 36369389 PMC10921989

[10] Ravaud A Motzer RJ Pandha HS George DJ Pantuck AJ Patel A . Adjuvant Sunitinib in high-risk renal-cell carcinoma after nephrectomy. N Engl J Med. (2016) 375:2246–54. doi: 10.1056/NEJMoa1611406 27718781

[11] Motzer RJ Russo P Haas N Doehn C Donskov F Gross-Goupil M . Adjuvant Pazopanib versus placebo after nephrectomy in patients with localized or locally advanced renal cell carcinoma: Final overall survival analysis of the phase 3 PROTECT trial. Eur Urol. (2021) 79:334–8. doi: 10.1016/j.eururo.2020.12.029 33461782 PMC8407394

[12] Haas NB Manola J Uzzo RG Flaherty KT Wood CG Kane C . Adjuvant sunitinib or sorafenib for high-risk, non-metastatic renal-cell carcinoma (ECOG-ACRIN E2805): a double-blind, placebo-controlled, randomised, phase 3 trial. Lancet. (2016) 387:2008–16. doi: 10.1016/S0140-6736(16)00559-6 26969090 PMC4878938

[13] Eisen T Frangou E Oza B Ritchie AWS Smith B Kaplan R . Adjuvant Sorafenib for renal cell carcinoma at intermediate or high risk of relapse: Results from the SORCE randomized phase III intergroup trial. J Clin Oncol Off J Am Soc Clin Oncol. (2020) 38:4064–75. doi: 10.1200/JCO.20.01800 33052759 PMC7768344

[14] Gross-Goupil M Kwon TG Eto M Ye D Miyake H Seo SI . Axitinib versus placebo as an adjuvant treatment of renal cell carcinoma: results from the phase III, randomized ATLAS trial. Ann Oncol Off J Eur Soc Med Oncol. (2018) 29:2371–8. doi: 10.1093/annonc/mdy454 30346481 PMC6311952

[15] Allaf ME Kim S-E Master V McDermott DF Harshman LC Cole SM . Perioperative nivolumab versus observation in patients with renal cell carcinoma undergoing nephrectomy (PROSPER ECOG-ACRIN EA8143): an open-label, randomised, phase 3 study. Lancet Oncol. (2024) 25:1038–52. doi: 10.1016/S1470-2045(24)00211-0 38942046 PMC11323681

[16] Motzer RJ Russo P Grünwald V Tomita Y Zurawski B Parikh O . Adjuvant nivolumab plus ipilimumab versus placebo for localised renal cell carcinoma after nephrectomy (CheckMate 914): a double-blind, randomised, phase 3 trial. Lancet. (2023) 401:821–32. doi: 10.1016/S0140-6736(22)02574-0 36774933 PMC10259621

[17] Pal SK Uzzo R Karam JA Master VA Donskov F Suarez C . Adjuvant atezolizumab versus placebo for patients with renal cell carcinoma at increased risk of recurrence following resection (IMmotion010): a multicentre, randomised, double-blind, phase 3 trial. Lancet. (2022) 400:1103–16. doi: 10.1016/S0140-6736(22)01658-0 36099926

[18] Choueiri TK Tomczak P Park SH Venugopal B Ferguson T Symeonides SN . Overall survival with adjuvant pembrolizumab in renal-cell carcinoma. N Engl J Med. (2024) 390:1359–71. doi: 10.1056/NEJMoa2312695 38631003

[19] Powles T Tomczak P Park SH Venugopal B Ferguson T Symeonides SN . Pembrolizumab versus placebo as post-nephrectomy adjuvant therapy for clear cell renal cell carcinoma (KEYNOTE-564): 30-month follow-up analysis of a multicentre, randomised, double-blind, placebo-controlled, phase 3 trial. Lancet Oncol. (2022) 23:1133–44. doi: 10.1016/S1470-2045(22)00487-9 36055304

[20] Choueiri TK Motzer RJ Karam JA Ye D He Z Caglevic C . Adjuvant pembrolizumab plus belzutifan versus pembrolizumab for clear cell renal cell carcinoma (ccRCC): The randomized phase 3 LITESPARK-022 study. J Clin Oncol. (2026) 44:LBA418–LBA418. doi: 10.1200/JCO.2026.44.7_suppl.LBA418 42586871

[21] Oza B Frangou E Smith B Bryant H Kaplan R Choodari-Oskooei B . RAMPART: A phase III multi-arm multi-stage trial of adjuvant checkpoint inhibitors in patients with resected primary renal cell carcinoma (RCC) at high or intermediate risk of relapse. Contemp Clin Trials. (2021) 108:106482. doi: 10.1016/j.cct.2021.106482 34538402 PMC8520913

[22] Roldan-Romero JM Santos M Lanillos J Caleiras E Anguera G Maroto P . Molecular characterization of chromophobe renal cell carcinoma reveals mTOR pathway alterations in patients with poor outcome. Mod Pathol. (2020) 33:2580–90. doi: 10.1038/s41379-020-0607-z 32616874

[23] Nickerson ML Warren MB Toro JR Matrosova V Glenn G Turner ML . Mutations in a novel gene lead to kidney tumors, lung wall defects, and benign tumors of the hair follicle in patients with the Birt-Hogg-Dubé syndrome. Cancer Cell. (2002) 2:157–64. doi: 10.1016/S1535-6108(02)00104-6 12204536

[24] Hasumi Y Baba M Ajima R Hasumi H Valera VA Klein ME . Homozygous loss of BHD causes early embryonic lethality and kidney tumor development with activation of mTORC1 and mTORC2. Proc Natl Acad Sci USA. (2009) 106:18722–7. doi: 10.1073/pnas.0908853106 19850877 PMC2765925

[25] Michalaki V Gennatas C . Chromophobe renal cell carcinoma with prolonged response to targeted therapy: a case report. J Med Case Rep. (2012) 6:115. doi: 10.1186/1752-1947-6-115 22524151 PMC3350425

[26] Davis CF Ricketts CJ Wang M Yang L Cherniack AD Shen H . The somatic genomic landscape of chromophobe renal cell carcinoma. Cancer Cell. (2014) 26:319–30. doi: 10.1016/j.ccr.2014.07.014 25155756 PMC4160352

[27] Ryan CW Tangen CM Heath EI Stein MN Meng MV Alva AS . Adjuvant everolimus after surgery for renal cell carcinoma (EVEREST): a double-blind, placebo-controlled, randomised, phase 3 trial. Lancet. (2023) 402:1043–51. doi: 10.1016/S0140-6736(23)00913-3 37524096 PMC10622111

[28] Gulati S Tangen C Ryan CW Vaishampayan UN Shuch BM Barata PC . Adjuvant everolimus in non–clear cell renal cell carcinoma: A secondary analysis of a randomized clinical trial. JAMA Netw Open. (2024) 7:e2425288. doi: 10.1001/jamanetworkopen.2024.25288 39106067 PMC11304111

[29] Koh Y Lim HY Ahn JH Lee J-L Rha SY Kim YJ . Phase II trial of everolimus for the treatment of nonclear-cell renal cell carcinoma. Ann Oncol. (2013) 24:1026–31. doi: 10.1093/annonc/mds582 23180114

[30] Koshkin VS Barata PC Zhang T George DJ Atkins MB Kelly WJ . Clinical activity of nivolumab in patients with non-clear cell renal cell carcinoma. J Immunother Cancer. (2018) 6:9. doi: 10.1186/s40425-018-0319-9 29378660 PMC5789686

[31] McDermott DF Lee J-L Ziobro M Suarez C Langiewicz P Matveev VB . Open-label, single-arm, phase II study of pembrolizumab monotherapy as first-line therapy in patients with advanced non-clear cell renal cell carcinoma. J Clin Oncol Off J Am Soc Clin Oncol. (2021) 39:1029–39. doi: 10.1200/JCO.20.02365 33529058 PMC8078262

[32] Hutson TE Michaelson MD Kuzel TM Agarwal N Molina AM Hsieh JJ . A single-arm, multicenter, phase 2 study of lenvatinib plus everolimus in patients with advanced non-clear cell renal cell carcinoma. Eur Urol. (2021) 80:162–70. doi: 10.1016/j.eururo.2021.03.015 33867192 PMC12684810

[33] Voss MH Molina AM Chen Y-B Woo KM Chaim JL Coskey DT . Phase II trial and correlative genomic analysis of everolimus plus bevacizumab in advanced non–clear cell renal cell carcinoma. J Clin Oncol. (2016) 34:3846–53. doi: 10.1200/JCO.2016.67.9084 27601542 PMC5791841

[34] Lee C-H Voss MH Carlo MI Chen Y-B Zucker M Knezevic A . Phase II trial of cabozantinib plus nivolumab in patients with non–clear-cell renal cell carcinoma and genomic correlates. J Clin Oncol. (2022) 40:2333–41. doi: 10.1200/JCO.21.01944 35298296 PMC9287282

[35] Grünwald V Bex A Rottey S Suárez C Procopio G Velasco G . Current status of adjuvant immunotherapy and relapse management in renal cell carcinoma: Insights from a European delphi study. Eur J Cancer. (2025) 225:115569. doi: 10.1016/j.ejca.2025.115569 40516320

[36] Tran J Ornstein MC . Clinical review on the management of metastatic renal cell carcinoma. JCO Oncol Pract. (2022) 18:187–96. doi: 10.1200/OP.21.00419 34529499

[37] U. F. a. D . US food and drug administration website. Silver Spring, Maryland: U.S. Food and Drug Administration (FDA). (2021).

[38] Keytruda (2018). European Medicines Agency (EMA. Available online at: https://www.ema.europa.eu/en/medicines/human/EPAR/keytruda (Accessed August 12, 2026).

[39] Kidney Cancer: Nice Guideline Expert Insight. Available online at: https://reference.medscape.com/cc1/p10/renal-cell-carcinoma-trials-can-inform-nice-guideline-kidney-2024a1000h4w#7 (Accessed August 13, 2026).

[40] FDA . FDA approves belzutifan with pembrolizumab for adjuvant treatment of renal cell carcinoma (2026). FDA. Available online at: https://www.fda.gov/drugs/resources-information-approved-drugs/fda-approves-belzutifan-pembrolizumab-adjuvant-treatment-renal-cell-carcinoma (Accessed June 14, 2026).

